# Induction of Conjugation and Zygospore Cell Wall Characteristics in the Alpine *Spirogyra mirabilis* (Zygnematophyceae, Charophyta): Advantage under Climate Change Scenarios?

**DOI:** 10.3390/plants10081740

**Published:** 2021-08-23

**Authors:** Charlotte Permann, Klaus Herburger, Martin Felhofer, Notburga Gierlinger, Louise A. Lewis, Andreas Holzinger

**Affiliations:** 1 Department of Botany, Functional Plant Biology, University of Innsbruck, 6020 Innsbruck, Austria; Charlotte.Permann@uibk.ac.at; 2 Section for Plant Glycobiology, Department of Plant and Environmental Sciences, University of Copenhagen, 1871 Frederiksberg, Denmark; klaus.herburger@plen.ku.dk; 3 Department of Nanobiotechnology, University of Natural Resources and Life Sciences Vienna (BOKU), 1190 Vienna, Austria; martin.felhofer@boku.ac.at (M.F.); burgi.gierlinger@boku.ac.at (N.G.); 4 Department of Ecology and Evolutionary Biology, University of Conneticut, Storrs, CT 06269-3043, USA; louise.lewis@uconn.edu

**Keywords:** alpine region, cell wall, conjugation, *Spirogyra*, sexual reproduction, streptophyte, zygospore

## Abstract

Extreme environments, such as alpine habitats at high elevation, are increasingly exposed to man-made climate change. Zygnematophyceae thriving in these regions possess a special means of sexual reproduction, termed conjugation, leading to the formation of resistant zygospores. A field sample of *Spirogyra* with numerous conjugating stages was isolated and characterized by molecular phylogeny. We successfully induced sexual reproduction under laboratory conditions by a transfer to artificial pond water and increasing the light intensity to 184 µmol photons m^−2^ s^−1^. This, however was only possible in early spring, suggesting that the isolated cultures had an internal rhythm. The reproductive morphology was characterized by light- and transmission electron microscopy, and the latter allowed the detection of distinctly oriented microfibrils in the exo- and endospore, and an electron-dense mesospore. Glycan microarray profiling showed that *Spirogyra* cell walls are rich in major pectic and hemicellulosic polysaccharides, and immuno-fluorescence allowed the detection of arabinogalactan proteins (AGPs) and xyloglucan in the zygospore cell walls. Confocal RAMAN spectroscopy detected complex aromatic compounds, similar in their spectral signature to that of *Lycopodium* spores. These data support the idea that sexual reproduction in Zygnematophyceae, the sister lineage to land plants, might have played an important role in the process of terrestrialization.

## 1. Introduction

Zygnematophyceae are a class of widespread and globally distributed streptophytic green algae. Recently, they have been resolved as the immediate sister lineage to land plants [1,2,3] and the first three genomes of Zygnematophyceae have been published [4,5]. They occupy various environments, including extreme habitats at high altitudes and/or latitudes, and belong to the most common primary producers in Arctic and Antarctic regions [6,7,8,9,10]. Numerous studies on abiotic stress tolerance and molecular mechanisms essential for surviving in terrestrial habitats have been undertaken. These studies conclude that Zygnematophyceae are well adapted to abiotic stresses, such as short growing seasons, high irradiation, and limited water availability [11,12,13,14,15,16,17,18]. Extended snow cover is a protector of the alpine vegetation against these stresses; however, it limits the duration of the growing season. Vegetative adaptations of Zygnematophyceae, such as formation of pre-akinetes [19,20], would most likely be beneficial during global warming, which can increase the frequency and intensity of theses stresses. Because climate change is projected to be more pronounced in high mountains and polar regions, other adaptations involving sexual reproduction may be critical for survival during increasingly severe temperature and desiccation stresses.

To date, the majority of stress research has focused on the physiology and cell biology of Zygnematophyceae in their vegetative state. In contrast, the stress physiology and biochemistry of the sexual stages of Zygnematophyceae remain comparatively underinvestigated. In most plants, the reproductive structures are more vulnerable than the vegetative cells, posing potential difficulties in extreme environments. Sexual reproduction in Zygnematophyceae, termed conjugation, has rarely been observed in samples from polar regions [8,21], although the protective and resistant structure of the resulting zygospores may be beneficial for withstanding the conditions of harsh environments. One reason for the rarity of this observation may be that sexual reproduction is an investment that requires a relatively long period for the ripening of zygospores and their complicated walls. Because evolutionary studies revealed the position of Zygnematophyceae as the closest relatives to land plants, conjugation most likely represents a crucial factor to enable terrestrialization [2].

Conjugation involves the fusion of two aplanogametes (gametes lacking organelles for locomotion) via a conjugation tube, and their development into a zygospore [22]. Although this process does not necessarily involve liquid water as a medium, it usually takes place in moist habitats [22]. Based on the cells involved in this process, a distinction between scalariform and lateral/terminal conjugation can be made. The term scalariform is applied when two gametes from different filaments fuse in the conjugation tube, resulting in a ladder-like appearance. Lateral or terminal conjugation occurs within one single filament, and the difference is due to the origin of the conjugation tube. In each case, further subdivisions can be made based on the location of gamete fusion and zygospore formation. The resulting zygospores also differ in shape, color, and structure, and exhibit a special, complex cell wall. The basic zygospore wall structure consists of three layers, termed endo-, meso-, and exospore [22]. Although the inner and outer layers (exo- and endospores) are usually colorless and composed of polysaccharides, the blue or brown mesospore is hypothesized to contain sporopollenin-like material, possibly algaenan [23,24]. The massive and complex zygospore wall, due to its biochemically generated “water-proof” layers, may be essential for these algae to tolerate climatic extremes, such as high irradiation or water scarcity leading to desiccation. The classical zygnematophycean taxonomy is heavily based on the morphological characteristics of sexual reproduction, explaining why most studies on zygospores have focused on morphological traits [25,26,27,28]. To date, little is known about the ultrastructural and biochemical features of the zygospore walls of Zygnematophyceae.

In the field, conjugation is rather rare in some species and often subjected to seasonality, making the cultivation of sexually reproducing Zygnematophyceae critical for future research. Unfortunately, the induction of sexual reproduction is difficult, because many internal and external factors are involved [29,30,31,32,33]. Some cultures established from field samples are also thought to lose their ability for sexual reproduction as they adapt to culture conditions [34]. Consequently, induction of conjugation under laboratory conditions has only been successful in a few genera, such as *Spirogyra* [35,36,37,38,39]. Zwirn et al. [39] successfully performed conjugation in nine of 95 *Spirogyra* strains collected from various European locations. Only recently, Takano et al. [38] induced zygospores or aplanospores in 15 of 52 *Spirogyra* strains originating from Japan. There are other *Spirogyra* isolates (*S. pratensis*, MZCH10213) for which the successful induction of sexual reproduction under laboratory conditions has been described [40]; however, the continuous induction of sexual reproduction remains difficult (H. Buschmann, Osnabrück & B. Classen, Kiel; personal communication).

There is an increasing interest in hallmarks of zygnematophycean terrestrialization, with some evidence from *Spirogyra* transcriptomes. The first experimental approaches exposing vegetative *Spirogyra* cells to elevated temperatures (37 °C) led to drastic transcriptomic and metabolic shifts [41]. Another interesting observation is that compounds, such as ethylene, appear to be a conserved phytohormone in *Spirogyra* [42,43], with a major impact on vegetative cell wall extensibility. The mechanism is likely realized by modifications of the cell wall matrix by expansins and xyloglucan endotransglucosylases/hydrolases [43]. Although the cell wall biochemistry is reasonably well studied in vegetative *Spirogyra* [44,45], and individual components such as xyloglucans [46] or arabinogalactan proteins (AGPs) [47] have been detected, few studies have addressed the cell wall composition of *Spirogyra* during sexual reproduction [36,48]. Interestingly, *Spirogyra* is the only genus for which evidence of the occurrence of algaenen, a resistant biomacromolecule, in zygnematophycean zygospores has been described to date [49].

**Figure 1:**
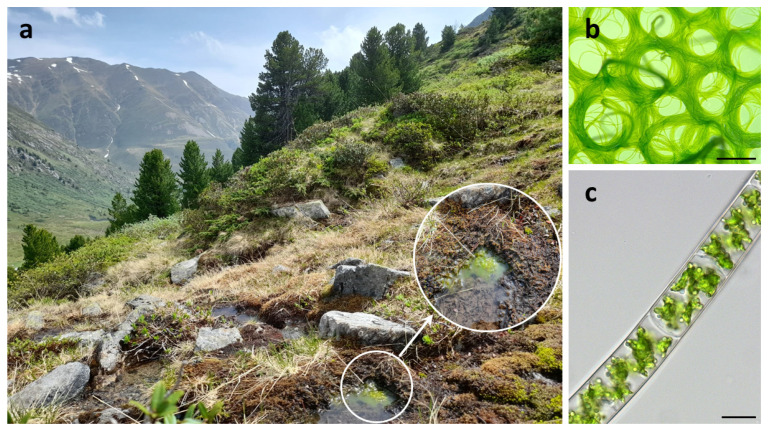
Habitat of isolated *Spirogyra mirabilis*: (**a**) sampling site with zygnematophycean green algal mat, (**b**) isolated unialgal culture, (**c**) detailed view of vegetative filament. Scale bars (**b**) 1 mm, (**c**) 20 µm.

In this study, we collected samples of *Spirogyra* that were rich in conjugation stages and zygospores from the Austrian Alps (Figure 1a–c), and from these we established a unialgal culture. We hypothesized that: (A) isolates established from field material exhibiting abundant sexual reproduction stages should be capable of sexual reproduction under appropriate laboratory conditions too; (B) the mesospore of mature zygospores contains aromatic compounds similar to those observed in other zygnematopyhcean zygospores [23,24]; and that (C) certain cell wall compounds (e.g., AGPs) known to play a vital role in reproductive structures of flowering plants and evident in *Spirogyra* vegetative cells ([47], B. Classen, Kiel; personal communication) can also be found in the zygospore wall. The initial morphological species determination was followed by a multi-gene phylogenetic analysis, which allowed to determine the isolated strain and gain new insights into the rarely observed sexual reproduction. The conjugation process and zygospore formation was documented by light- and fluorescence microscopy, and transmission electron microscopy (TEM), after high pressure freeze fixation/freeze substitution to reveal the ultrastructure of this process. Moreover, Comprehensive Microarray Polymer Profiling (CoMMP) was performed to obtain an overview of the relative abundance of cell wall components and confocal Raman spectroscopy enabled the analysis of the composition and distribution of carbohydrates, lipids, proteins, and aromatic residues in the zygospore. Our data contribute to the increasing knowledge on sexual reproduction in Zygnematophyceae. Because zygospores are designed to tolerate unfavorable abiotic conditions, this knowledge is important to understand how these algae adapted to semi-terrestrial habitats.

## 2. Results

### 2.1. Morphological Observations on Field Sampled Spirogyra sp. and Establishment of Cultures

**Figure 2:**
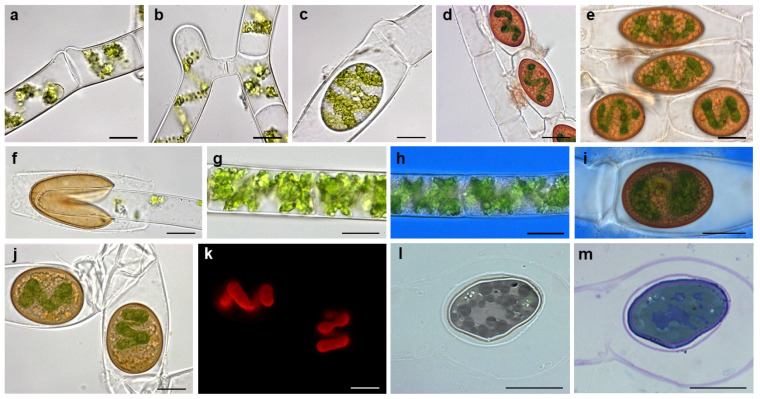
Conjugation and zygospores of *Spirogyra mirabilis*: (**a**) lateral conjugation, (**b**) scalariform conjugation, (**c**) zygospore formed by lateral conjugation, (**d**) zygospores formed by scalariform conjugation, (**e**) polymorphic zygospores, (**f**) germinating zygospore, (**g**) vegetative filament, (**h**) vegetative filament stained with Indian ink, (**i**) zygospore stained with Indian ink, (**j**) zygospores with chloroplast with (**k**) corresponding chlorophyll autofluorescence image, (**l**) semi-thin section with (**m**) corresponding toluidine staining. Scale bars 20 µm.

Field-sampled *Spirogyra* sp. showed an anisogamous monogametangial conjugation that was scalariform and lateral (Figure 2a–d). They formed brown, polymorphic zygospores ranging from spherical to lenticular in shape, and exhibited a smooth surface structure (Figure 2e). Germinating zygospores showing a clear split of the zygospore wall into two halves were occasionally found in stored field samples 10 months after sampling (Figure 2f). The vegetative filaments and the gametangia containing the zygospore were not surrounded by a mucilage sheath, as known for other Zygnematophyceae (Figure 2g–i). Although the wall of the freshly formed zygospores turned brown within a few days, the chloroplasts were still visible and chlorophyll autofluorescence was detectable (Figure 2j,k). Semi-thin sections stained with toluidine blue, predominantly staining acidic polysaccharides, showed a clear lilac coloration of the gametangium cell wall—a layer encasing the zygospore—and the inner layer of the zygospore wall (Figure 2l,m). The outer zygospore wall exhibited a stronger purple stain (Figure 2l,m).

### 2.2. Conjugation in Spirogyra mirabilis Can Be Induced under Laboratory Conditions, but May Be Dependent on Internal Factors 

When cultivated on BBM plates under standard cultivation conditions, the filaments showed no sign of conjugation. After being placed on APW under considerably higher PAR (from ~30 to 184 µmol photons m^−2^ s^−1^) and continuous light, sexual reproduction began about 11 days after inoculation in six plates (Supplementary Figure S1). The sexual reproduction generated in the laboratory also led to scalariform and lateral conjugation. Usually, the brown coloration of the zygospore wall developed only a few days after their formation. Although laboratory-induced conjugation was successfully performed in early spring, no conjugation was observed under the same cultivation conditions later in the season, indicating that some internal factor exists controlling this process.

### 2.3. Phylogenetic Position of the Investigated Spirogyra

We obtained a partial *rbc*L sequence from the isolated culture of *Spirogyra* sp. (Kühtai) with a length of 1313 bp, and a partial *atp*B sequence with a length of 873 bp, and the sequences were deposited in the NCBI GenBank (*rbc*L sequence: MZ813178; *atp*B sequence: MZ813179). 

**Figure 3:**
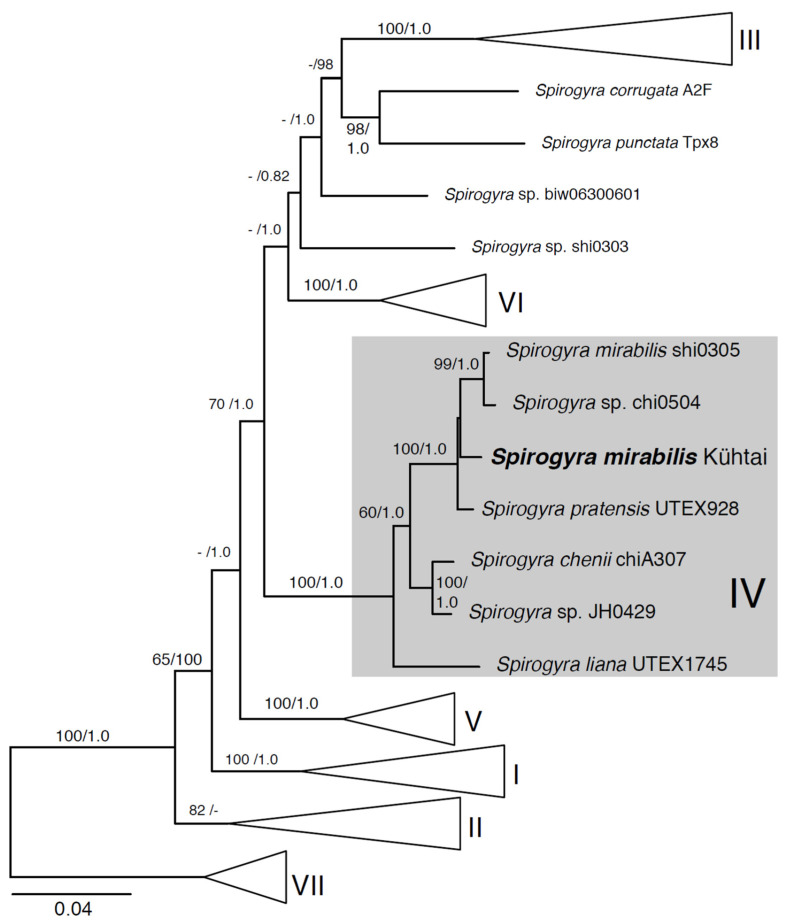
Summary of the maximum likelihood analysis of *rbc*L and *atp*B sequences from *Spirogyra mirabilis* (Kühtai) in the context of related and sequenced *Spirogyra* strains in clade IV, and for which the full tree is presented in Supplementary Figure S2. The tree is oriented, and the major clades are labeled, after Stancheva et al. [27] and Takano et al. [38]. Node support values correspond to ML bootstrap/BPP values. Scale bar indicates the expected number of substitutions/sites.

Both the ML and BI analyses of the combined *rbc*L and *atp*B gene sequences placed the investigated *Spirogyra* sp. (Kühtai) within clade IV of *Spirogyra* sp. [27,38], closely affiliated with *S. mirabili*s (NIES), *S. pratensis* (UTEX 928), and *Spirogyra* sp. chi0504 (see the overview tree, Figure 3, and the full tree summarizing the ML and BI analyses in Supplementary Figure S2). Clade IV *Spirogyra* taxa were separated from strains designated as *S. longata* by Takano et al. [38] (in clade I, Figure S2, arrowhead), which share some morphological features with *S. mirabilis* and *S. pratensis*. Based on the conjugation morphology and the vegetative filament characteristics, the sample was first determined to be *Spirogyra longata.* When comparing the morphological characters of the isolate with *S. mirabilis* and the closely positioned *S. pratensis*, we found numerous overlaps (Supplementary Table S1). However, the greatest number of conformities was found with *S. mirabilis*, corresponding to our phylogenetic results (Supplementary Table S1).

### 2.4. Spirogyra Zygospore Ultrastructure

**Figure 4:**
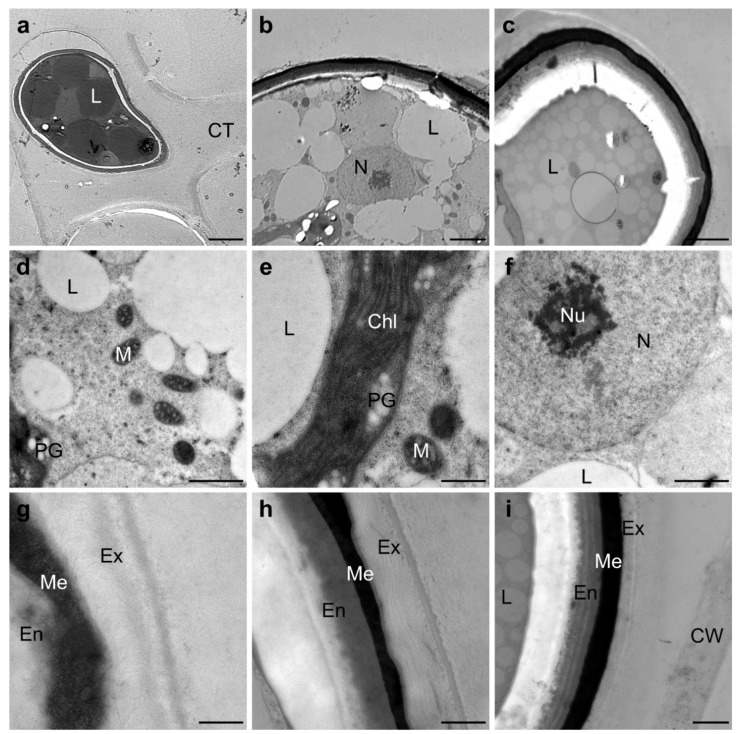
Transmission electron micrographs of zygospores of *Spirogyra mirabilis*: (**a**) gametangia with zygospore and conjugation tube, (**b**) overview of mature zygospore, (**c**) mature zygospore with lipid accumulation, (**d**) mitochondria and lipid bodies in the cell lumen, (**e**) detailed view of the chloroplast lobes with plastoglobuli, (**f**) nucleus with nucleolus, (**g**) detailed view of the zygospore wall of a presumably young zygospore with visible exospore structure, (**h**) mature zygospore wall, (**i**) mature zygospore with distinct orientation of the microfibrils of the endospore. Abbreviations: Chl—chloroplast, CT—conjugation tube, CW—cell wall of the gametangium, En—endospore, Ex—exospore, L—lipid bodies, Me—mesospore, M—mitochondrion, N—nucleus, Nu—nucleolus, PG—plastoglobuli. Scale bars (**a**) 5 µm, (**b**,**c**) 2500 nm, (**d**,**f**,**i**) 1 µm, (**e**,**g**,**h**) 500 nm.

Ultrastructural investigations by TEM showed a high abundance of storage compounds, mostly as lipid bodies in the cell lumen of the zygospores (Figure 4a–c). Cell organelles such as mitochondria and chloroplasts with plastoglobuli were still present in some cells, whereas these components were reduced in others (Figure 4d,e). A nucleus with nucleolus was also present in the zygospores (Figure 4f). The zygospore wall was composed of three main layers, the endo-, meso-, and exospore (Figure 4g–i). The mesospore was the most electron-dense component, followed by the endospore, whereas the exospore exhibited a looser structure (Figure 4h). Detailed views of the zygospore wall showed an intertwining or parallel orientation of the microfibrils in the outer layer (Figure 4g,h). In contrast, the architecture of the endospore appeared to be clearly layered with a pronounced orientation of the microfibrils (Figure 4i).

### 2.5. Spirogyra Cell Walls Are Rich in Pectins, Hemicelluloses, and Glycoproteins

**Figure 5:**
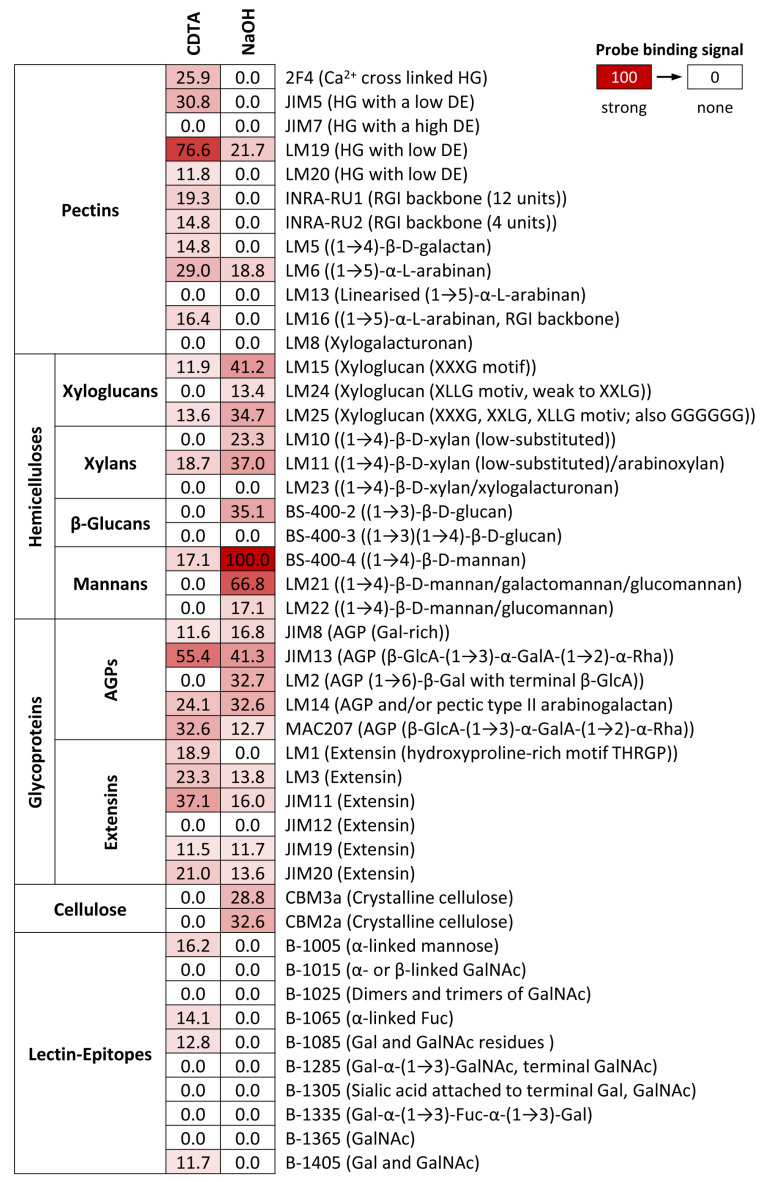
Determining cell wall epitopes in *Spirogyra mirabilis* using carbohydrate microarray profiling. A total of >40 cell wall probes were incubated with CDTA and NaOH extracts sequentially prepared from Spirogyra AIR. The heatmap color intensity shows the strength of the probe binding. The strongest signal was assigned a value of 100 and the cut-off signal was set to 5. Probe codes are in bold and bound epitopes in brackets. Abbreviations: Ara—arabinose, DE—degree of esterification, Fuc—fucose, Gal—galactose, GalA—galacturonic acid, GalNAc—N-acetylgalactosamin, GlcA—glucuronic acid, HG—homogalacturonan, RGI—rhamnogalacturonan I, Rha—rhamnose, AGPs—arabinogalactan proteins. Oligosaccharide nomenclature for xyloglucan probes, see Fry et al. [50].

We evaluated the polysaccharide and glycoprotein composition of *S. mirabilis* cell walls via CoMPP to test for the presence of 46 cell wall epitopes. Samples contained both the vegetative (filaments) and generative (zygospores) stage, allowing us to gain a broad insight into the algal cell wall composition. CDTA, an excellent solvent for pectin, extracted high amounts of homogalacturonan (HG), where LM19 (binding to HG with low DE) showed the strongest signal, followed by JIM5 and 2F4, which recognize similar epitopes (Figure 5). LM20 and JIM7, binding to higher methylated HG, produced a signal that was low or below the threshold, respectively (Figure 5). Higher signals were found for probes recognizing rhamnogalacturonan I (RGI; probes INRA-RU1+2, LM16), and linear arabinans (LM6; Figure 5). In addition to pectin, the second major class of cell wall components in the CDTA fraction was glycoproteins, yielding high binding of probes that recognize different motifs on arabinogalactan proteins (AGPs; JIM13, MAC207, LM14) and extensins (JIM11, LM3, JIM20, LM1, JIM13). Apart from LM1, which binds to a hydroxyproline-rich AGP motif, the mentioned extensin probes also delivered appreciable signals for the NaOH extraction fraction (Figure 5). Interestingly, we also found binding of some lectin probes in the CDTA fraction; these probes recognize mannose (B-1005), fucose (B-1065), galactose, and N-acetylgalactosamin residues (B-1085, B-1405; Figure 5). The NaOH fraction was dominated by beta-glycans, particularly mannans, where probes against linear mannans (BS-400-4) and hetero-mannans (LM21) delivered the strongest signals (Figure 5). Furthermore, we found abundant epitopes of xyloglucans (LM15, LM25), xylans (LM10, LM11), and callose (BS-400-2). The NaOH fraction also contained appreciable amounts of glycoproteins, dominated by AGPs. Interestingly, and in contrast to other AGP probes, LM2 only bound in the NaOH fraction (Figure 5). This probe recognizes galactose glycosylation motifs that contain terminal glucuronic acid. Finally, and as expected, cellulose probes (CBM2a, CBM2a) only delivered a signal for the NaOH fraction (Figure 5).

### 2.6. Immunostainings Reveal Arabinogalactan Proteins as Part of the Resistant Zygospore Wall

**Figure 6:**
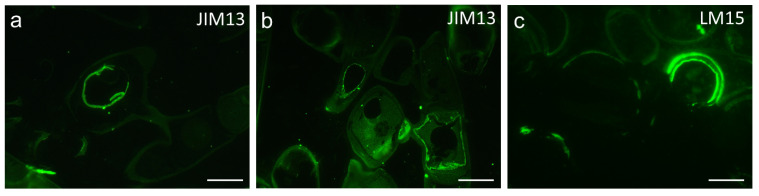
Immuno-localization of certain cell wall components in *Spirogyra mirabilis*. (**a**, **b**) JIM13 (AGP) and (**c**) LM15 (xyloglucan) of semi-thin sections of *Spirogyra mirabilis*, (**a**) zygospore in gametangium, (**b**) conjugating cells, (**c**) bilayer arrangement in zygospores wall. Scale bars 20 µm.

Semi-thin sections of zygospores established from cultures produced a strong signal in the cell wall when stained with JIM13 (AGP) and occasionally with LM15 (xyloglucan) (Figure 6a–c). JIM13 also produced a signal in a conjugating gametangia, whereas little signal was found in the old conjugation tube (Figure 6a,b). Almost no signal was found in cells or zygospores when stained with the antibodies LM19 (partially methylesterified HG), BS-400-4 ((1->4)- β -D-mannan), LM1 (extensin), LM25 (xyloglucan), LM6 ((1->5) α-L-arabinan), JIM5 (HG with low degree of esterification (DE)), or LM10 ((1->4)-β-D-xylan)) (Supplementary Figure S3). The lack of signal after BS-400-4 staining of the sections was likely due to epitope masking. 

### 2.7. Confocal Raman Microscopy Detects Aromatic Compounds Similar to Lycopodium Spore Cell Walls

**Figure 7:**
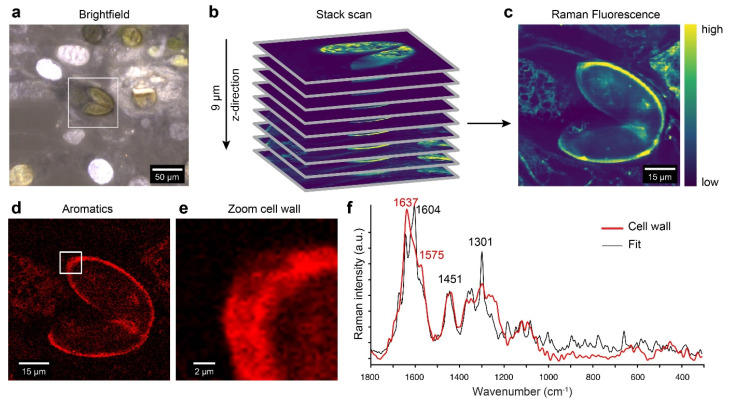
Raman imaging of *Spirogyra mirabilis* zygospores reveals aromatic compounds in the cell wall. (**a**) Brightfield image of the investigated spore (white rectangle). (**b**) Confocal Raman images with a depth profiling of 9 μm, measured as nine layers in a stack for every micrometer in the z-direction, up to down. (**c**) Raman fluorescence image of the middle layer of the spore. (**d**) Aromatic compounds impregnate the cell wall as visualized by integrating the peak at 1604 cm^−1^. (**e**) Magnification of the cell wall shows high intensity of aromatic compounds in the zygospore cell wall, but no clear layering. (**f**) The extracted average spectrum from the *Spirogyra* cell wall (red line) shows clear aromatic bands around 1600 cm^−1^. Fitting of reference Raman spectra by a linear combination using the Orthogonal Matching Pursuit Method confirmed the aromatic nature and placed the *Lycopodium* spore cell wall spectrum on top with the highest contribution (similarity) (Supplementary Figure S4).

The *Spirogyra* zygospores investigated by confocal Raman microscopy were stored in the refrigerator prior to investigation and showed a dark green to brown coloration and some had already germinated, as indicated by the separation of the two halves (Figure 7a). The brown pigmentation led to laser-induced high fluorescence or even burning of the sample. Therefore, we first performed a fast z-stack scan to bleach the entire spore and find the optimal focal plane (Figure 7b). The Raman fluorescence image of the innermost part showed the high intensity of the zygospore wall, pointing to a high content of aromatic compounds (Figure 7c) [51]. Integrating over the wavenumber range corresponding to aromatic compounds (1550—1705 cm^−1^) [52] confirmed the aromatic nature by visualizing the cell wall with the highest intensity (Figure 7d). Due to the short integration time, the spectra of the z-stack spectra were too noisy for a detailed analysis of the cell wall. Therefore, we focused on a small area of the zygospore cell wall and measured with higher laser power and longer integration time to obtain high-quality Raman spectra (Figure 7e). The extracted zygospore cell wall spectrum shows clear aromatic band contributions at 1637 cm^−1^ with shoulders at 1604 and 1575 cm^−1^, suggesting a complex aromatic nature (Figure 7f, red spectrum). To obtain insights into the components reflected by the cell wall spectrum, we applied the Orthogonal Matching Pursuit (OMP) method [53]. This algorithm searched a database of reference components (aromatics, lipids, and carbohydrates, *n* = 267) regarding coincidence and fits with a linear combination the cell wall spectrum using the most suitable (similar) reference spectra. We included a Raman spectrum of a *Lycopodium* spore cell wall in the database, and this was chosen in first place by the algorithm to model the average spectrum of the zygospore cell wall (Figure 7f, black line fit, Supplementary Figure S4 reference spectra included in the fit). In addition to the *Lycopodium* spore cell wall spectrum with main bands at 1604 and 1451 cm^−1^, flavanones and lipids were included in the fit to account for the bands at 1637, 1575, and 1296 cm^−1^ (Supplementary Figure S4b). The fit is not perfect, as not exactly the same cell wall as that in *Lycopodium* is present, and the fitted flavonoids and lipids may vary due to high diversity and interactions. However, a similar aromatic and lipidic nature of the cell wall as that of the cell wall of a *Lycopodium* spore can be concluded.

## 3. Discussion

In the present study we performed a comprehensive analysis of the phylogeny, morphology, ultrastructure, and cell wall composition of an alpine zygnematophycean species, *Spirogyra mirabilis*. The data suggest that the alga readily produces zygospores under laboratory conditions. Moreover, chemical analyses of the zygospore walls suggest that they contain—in addition to cellulose, AGPs, and hemicelluloses—complex aromatic compounds, thus their chemical composition resembles the cell walls of *Lycopodium* spores. These data strengthen our evolutionary view, suggesting that Zygnematopyhceae, the sister-group to land plants, are capable of producing highly resistant compounds, making zygospores ideal propagules to tolerate extreme scenarios caused by naturally occurring harsh environmental conditions or man-made climate change scenarios. 

### 3.1. Successful Induction of Conjugation in Spirogyra Isolates

We established a sexually reproducing culture of field-collected *Spirogyra* from the Austrian alps, a crucial step for studying the cell biology and physiology of sexual reproduction in species with strong seasonal reproduction. Establishing a culture also aids in accurate identification, given that field collected samples often contain hidden species diversity. Recent investigations of seven strains of arctic algae, which all exhibited a *Cylindrocystis* (Zygnematophyceae)-like morphology, revealed five different genotypes at the 18S rDNA level and seven different genotypes at the *rbc*L level [54]. Pichrtová et al. [21] studied the genetic variation of *Zygnema* filaments from the High Arctic and also found a surprisingly high diversity within communities, even though they contained vegetative filaments that were almost uniform morphologically.

The alpine *Spirogyra* samples investigated here easily adapted to cultivation, but underwent asexual reproduction when kept on BBM and at low PAR. However, growth on nutrient-poor APW medium and higher light levels (from PAR of 30 to 184 µmol photons m^−2^ s^−1^) induced conjugation, confirming results shown by Ikegaya et al. [36] and Takano et al. [38]. Nutrition, particulalrly nitrogen depletion and increased light intensity, are suspected to promote sexual reproduction in Zygnematopyhceae [24,32,39]. Previous studies also indicate that nitrogen depletion induces the formation of secondary carotenoids and sporopollenin synthesis, and may play a vital role in the differentiation of the gametangia [31,33]. A study conducted on 95 strains of *Spirogyra* found that the nutrition ratio in dependence of specific ecological demands is important, but no general triggers for sexual reproduction were found [39]. Yoon et al. [48], for example, reported sexual reproduction in *Spirogyra varians* cultivated on BBM medium at >20 µmol photons m^−2^ s^−1^, a comparatively nutrient-rich medium and low PAR. This supports the concept that conjugation is induced by a range of environmental factors. In addition to the external factors required for sexual reproduction in *S. mirabilis*, we also encountered the potential involvement of internal factors. Although *S.* *mirabilis* conjugated in the laboratory in early spring, no sexual reproduction was observed during summer. Czuda [25], who has made a significant contribution to the research of sexual reproduction in Zygnematophyceae, also encountered the possibility of internal factors involved in the successful induction of conjugation. This physiological condition is described to be sensitive to ostensible insignificant side influences and to subside within 3–5 days if the required external conditions for conjugation are not provided. Another study conducted on the life cycle and genetics of *Zygnema* also mentioned that conjugation might depend on internal factors [29]. Although both studies were conducted on *Zygnema circumcarinatum*, the possibility of the general involvement of a physiological condition in the sexual reproduction process of Zygnematophyceae cannot be dismissed, as there are few reports of continuously conjugating cultures. In the present study, such factors might manifest themselves in the form of seasonality. The original habitat of the collected sample often exhibits early cold snaps, whereby late zygospore formation may be too risky for some species. Kim and Kim [34], however, suspected that some cultures simply lose their ability for conjugation in the process of adapting to laboratory conditions, which also cannot be excluded in the present study.

### 3.2. Phylogenetic Analysis Contradicts Initial Morphological Species Assignment

The species-rich genus *Spirogyra* is thought to show signficant hidden diversity, due to high diversity seen during the analysis of SSU rRNA [55]. However, Gontcharov et al. [56] reported that *Spirogyra* was considered to be fast evolving in the SSU rRNA gene. Therefore, we followed the approaches of Stancheva et al. [27] and Takano et al. [38] and analyzed the *rbc*L and the *atp*B genes for our phylogenies. 

Species assignment in Zygnematophyceae is largely based on the reproductive morphology, because vegetative filaments only allow a specification at the genus level. Stancheva et al. [26] suggested that the mesospore color in *Zygnema* (Zygnematophyeae) represents an important criterion in the infrageneric classification of this genus. Our initial morphological species assignment (*S. longata*) could not be confirmed by the phylogenetic analysis of the *rbc*L and *atp*B genes. As mentioned earlier, a molecular approach for species assignment is important when working with sterile filaments; however, it was found that it is also crucial in samples of *Spirogyra* where the conjugating morphology is evident. When assigning the species on a morphological basis of the reproductive characteristics, the isolate was determined as *S. longata*, which shares many of the observed traits. Because *S. longata* has a polymorph zygospore morphology and the occurrence of sexual reproduction between two different filaments (scalariform) and adjacent cells (lateral) has been described, one may be tempted to classify the isolate examined here to this species. The phylogenetic analysis has shown that the isolate belongs to a different clade (clade IV according to Stancheva et al. [27], also confirmed by Takano et al. [38]). This clade includes, inter alia, S. *mirabilis* and S. *pratensis*. All three species, in addition to the alpine *Spirogyra* sp. investigated in this study, are very similar in their morphological characters, complicating morphological species determination (Supplementary Table S1). However, the overall traits exhibited by the alpine *Spirogyra* sp. are consistent with the morphology of *S. mirabilis* based on the descriptions of Pascher [57], Prescott [58], and Kadlubowska [22] (Supplementary Table S1), and our phylogenetic analyses also support the assignment of *Spirogyra* sp. (Kühtai) to *S. mirabilis*. Takano et al. [38] designated strain shi0305 as *S. mirabilis*, based on comparisons to historical literature. We note that, according to Takano et al. [38], *Spirogyra* sp. chi0504 does not bear a species designation but also possesses many traits that are consistent with *S. mirabilis*. *Spirogyra* sp. chi0504 shares a *rbc*L haplotype of strain JPS025, so there is also support for this strain as *S. mirabilis*.

In this context, most literature describes *S. mirabilis* as a species in which conjugation is rarely observed [22]. Instead, most records describe the formation of asexually formed aplanospores [38,57]. Czurda [25] even claimed that this species has “lost” the ability to sexually reproduce and described failed attempts to form conjugation tubes. We also observed this in some filaments. Although in all described cases a scalariform conjugation was observed, the present study also documented zygospores formed by two adjacent cells, i.e., lateral conjugation. Because the observed conjugation traits of *S. mirabilis* differ from the few existing descriptions, the current study may provide new important insights into this species. Geitler [59] mentioned that the species descriptions of *S. mirabilis* do not encompass the full range of variation observed, and also described rudimentary conjugation in some populations. However, it needs to be mentioned that a definite species determination cannot be made, due to the possibility of polyploidy and hybridization between different species. This complex variability in size and shape has been reported from various *Spirogyra* species, making any morphological species concept extremely difficult. The only way to overcome this problem is to perform a polyphasic approach, including not only morphological and molecular data, but also ecophysiological traits [28]. Here we sampled in an alpine extreme habitat, suggesting that organisms reproducing sexually are well adapted to this environment.

### 3.3. The Cell Wall Contains Various Polysaccharides, Glycoproteins, and Aromatic Compounds 

Previous studies found that the cell wall composition of Zygnematophyceae resembles those of their descendants, the land plants [60,61,62,63], which underlines the algal ancestry of the latter [64]. Moreover, a recent study confirmed the presence of fucosylated xyloglucan in zygnematalean cell walls by both genetic and biochemical evidence [65]. This suggests that all of the major land plant cell wall polysaccharides predate the emergence of Embryophytes. Indeed, we found abundant binding of probes recognizing mannans, xyloglucans, xylans, homo-, and rhamnogalacturonan in *Spirogyra* cell walls. Mannans produced the strongest signal in our CoMPP setup, confirming previous studies that found them to be a major hemicellulose in Spirogyra [61] and widely distributed among charophytes [66]. CoMPP detects extracted cell wall components. In contrast, *in situ* staining hardly detected mannans (see below), which might be due to common masking effects by HG [67], which we detected abundantly in *Spirogyra* cell walls. The majority of HG was extracted via CDTA; however, an appreciable fraction was still detectable in the NaOH extract. This suggests that these HG polymers were firmly bound to hemicellulosic polysaccharides such as mannans, which underpins the proposed mannan masking by HG [62]. The detection of numerous cell wall components and their complex extraction behavior suggest that the cell wall composition of *Spirogyra* is chemically and structurally complex, which was also concluded recently for the closely related green alga *Mougoetia disjuncta* [23]. Although *Spirogyra* shares major cell wall traits with *Mougeotia*, some interesting differences remain. For example, in *S. mirabilis*, RGI was fully extractable with CDTA—suggesting limited physical interactions with hemicelluloses—whereas RGI epitopes in *M. disjuncta* were roughly equally present in CDTA and NaOH. Mannans were scarce in *M. disjuncta*, which contained a higher proportion of HG and callose when compared with *S. mirabilis*. Furthermore, binding of lectins was absent in *M. disjuncta* but detectable in *S. mirabilis* (B-1005, B-1065, B-1085, B1405). It is likely that these signals stemmed from the generative structure in the sample. Kim et al. [6], Yoon et al. [48], and Ikegaya et al. [36] found binding of *Ulex europaeus* agglutinin (B-1065), concanavalin A (B-1005), and *Ricinus communis* agglutinin (B-1085) to papillae that give rise to conjugation tubes in *S. varians*, suggesting the presence of α-linked mannose/fucose, galactose, and/or N-acetylgalactosamine. Correspondingly, in the present study we also detected binding of the lectin B-1405, which can also bind to galactose and/or N-acetylgalactosamine residues.

### 3.4. Prominent Role of AGPs and Extensins in Spirogyra

In addition to detecting various polysaccharides, we found strong binding of probes targeting AGP and extensin glycoproteins in *S. mirabilis* cell walls. In most cases, probes produced signals in both the CDTA and NaOH fraction, suggesting that the detected glycoproteins interact with various other cell wall components. Extensins can self-assemble to highly regular networks [68] and may have a role in mediating the assembly of pectin rich structures. As shown previously, CGA possess pectic structures that resemble land plant cell plates [62]. In particular, cross cell walls between individual cells of filamentous Zygnematophyceae are rich in newly secreted methylated HG typical for cell plates [23,69], and it is thus likely that extensins also support the formation of cell wall structures in Charophytes. Most probes targeting AGPs delivered strong signals, confirming their presence in CGA [61], where they may be crucial for cell adhesion to the substrate [47]. By immunofluorescence labelling, an antibody against AGPs (JIM 13) produced the strongest signal in the zygospore, and AGPs were also detected in actively conjugating gametangia, whereas they were absent in an old conjugation tube. AGPs are suggested to be involved in several steps of the reproduction process of flowering plants [70], but the exact function during conjugation remains to be elucidated. In contrast to our CoMPP analysis, immunofluorescence staining did not yield a signal for mannans; however, the absence of a signal does not indicate a complete absence of the epitope in the mature zygospore wall, e.g., due to masking effects. The occurrence of xyloglucans in some zygospore walls suggests their biochemical relationship to vegetative *Spirogyra* cell walls, where these compounds have been detected [46].

### 3.5. Complex Arrangement of the Zygospore Cell Wall Suggests Maximal Protection

In contrast to the relatively simple structure of the vegetative cell wall in *Spirogyra*, the zygospore wall is composed of three layers, the exo-, meso-, and endospore. Both the zygnematophycean exo- and mesospore contain different types of polysaccharides [23,24]. Although the inner layer exhibited no distinct structure in two studied *Mougeotia* species [23], the endospore of mature *S. mirabilis* zygospores showed a clear layering and parallel orientation of the microfibrils (Figure 4i). This arrangement likely contributes to the mechanical strength of the zygospore cell wall in *Spirogyra*. In contrast, the exospore had a rather loose appearance, but still showed some texture. The different microfibril arrangements in the exo- and endospore between zygospores is likely due to different development stages, because the maturation process involves a massive reorganization of the zygospore wall. However, further TEM analyses are necessary to resolve this difference. In contrast, the mesospore exhibited high electron density in every zygospore observed, similar to that seen in the TEM investigations of *M. disjuncta* [23]. Because little is known regarding the chemical composition of the mesospore, we investigated the zygospore cell walls by Raman spectroscopy and revealed aromatic compounds that were similar in their chemical composition to those in *Lycopodium* spore cell walls. This can also be seen from an evolutionary perspective, where Zygnematopyhcae are closely related to land plants. Studies of two *Mougeotia* species also indicated an aromatic layer in the mesospore by Raman spectroscopy [23]. More specifically, the mesospore has been suggested to contain sporopollenin-like material, termed algaenan [23,24]. Algaenan is a highly aliphatic non-hydrolysable biomacromolecule found in microalgae [71]. Similar molecules occur in protective tissues of higher plants in the form of cutans or suberans [71]. We therefore hypothesize that the mesospore provides effective protection against water loss, another major abiotic stressor in extreme environments. The mesospore may also be crucial when zygospores are exposed to high irradiation, by acting as electron-dense bodies, presumably containing phenolic compounds, which have been suggested to be an important factor in UV protection in vegetative zygnematophycean cells [8,15,17]. This may be especially advantageous when living in habitats with high altitudes and/or latitudes, making sexual reproduction in these environments a key beneficial life stage.

## 4. Conclusions

The present study demonstrates the successful isolation and induction of conjugation of field-collected *Spirogyra*. It provides new information on the conjugation process of *S. mirabilis*, which is a rarely observed trait in this species. Discrepancies in the molecular and initial morphological species determination strengthen the importance of a polyphasic approach in species determination. Considering the chemical composition of the multilayered zygospore cell wall, our findings support the presence of AGPs, pectins, cellulose, hemicelluloses, and complex aromatic compounds, similar to those found in *Lycopodium* spores. The latter component, which is most likely located in the electron-dense mesospore, might be highly beneficial for survival in unfavorable habitats. Overall, sexual reproduction in Zygnematophyceae may be crucial for tolerating extreme abiotic stresses, such as elevated temperatures and desiccation, accompanying man-made climate change.

## 5. Material and Methods

### 5.1. Sampling Site/Plant Material and Isolation

Algal samples containing zygnematophycean filaments were taken in the Kühtai valley (Austria/Tyrol) in July 2020. The sampling site was a slow running rivulet near a main road at 2020 m.a.s.l. (47°21′76″ N, 11°03′77″ E, Figure 1a). The fresh samples were stored in water taken from the sampling site. Initial light microscopical investigations of the field samples were conducted one week after collecting. A unialgal culture isolate of a conjugating *Spirogyra* sp. with its typical morphology of the spiral chloroplast was established (Figure 1b,c), and then further characterized by phylogenetic analysis. Algal filaments were cultivated on Bold’s Basal Medium (BBM) [72], solidified on 1.8% agar plates, and incubated in a 16/8 h light-dark regime, 20/15 °C, and ~30 µmol photons m^−2^ s^−1^ photosynthetically active radiation (PAR, illuminated with cool white light; Osram Dulux L 55W/840). 

### 5.2. Induction of Conjugation

To induce sexual reproduction in *S. longata*, precultivated *Spirogyra* filaments were incubated at 184 µmol photons m^−2^ s^−1^ PAR under continuous light on agar plates containing Artificial Pond Water (APW, pH = 7) [38]. Plates were checked daily for conjugating stages via a dissecting microscope in a similar setting as that used by Ikegaya et al. [36] and Takano et al. [38].

### 5.3. Light-, Fluorescence, and Confocal Laser Scanning Microscopy 

Light micrographs were taken with a Zeiss Axiovert 200 M light microscope (Carl Zeiss AG, Jena, Germany), equipped with an Axiocam HRc camera (Carl Zeiss AG, Jena, Germany) and Zeiss Axiovision software. Indian ink (Dr. Ph. Martin’s, Oceanside, United States) staining was employed to detect potential mucilage sheaths in live cells (according to Permann et al. [23]). Chlorophyll auto fluorescence was visualized with a Zeiss Filter Set 09 (Excitation: band pass (BP) 450–490 nm and emission: long pass (LP) 515 nm); the same filter set was used for fluorescent antibody detection in immunocytochemistry. Confocal laser scanning microscopy (CLSM) was performed with a Zeiss Pascal system control using Zen 2009 software, and excitation with an argon laser 488 nm, and emission was collected at 505–550 nm (false colored green).

### 5.4. Phylogenetic Analysis

DNA extraction/PCR. DNA extraction of *Spirogyra* sp. (Kühtai) was performed using the ZymoBiomics DNA prep kit (ZymoResearch, Irvine CA, USA). PCR was performed using *rbc*L primers CHAR-RF-1, CHAR-RF-2, CHAR-RR-3, R42AE [73,74] and *atp*B primers *atp*B-175FZYG, *atp*B-700F, *atp*B-866R, *atp*B-1404RZYG [27]. Resulting PCR products were cleaned before sequencing with EXO-FastAP (Thermo Fisher Scientific, Waltham, MA, USA) using the manufacturer’s instructions. PCR products were sequenced with the appropriate forward and reverse PCR primers by a commercial sequencing facility using Sanger sequencing (Eurofins Genomics, LLC, Louisville, KY, USA).

Phylogenetic analysis. Newly generated sequences were added to the two-gene alignment obtained from Takano et al. [38] that had been trimmed to include only representatives of the focal genus *Spirogyra*. A table of the sequences used in this study is available (Supplementary Table S2). The resulting data matrix was analyzed using maximum likelihood (ML) and Bayesian inference (BI). The data were analyzed under the maximum likelihood criterion following the GTR+I+gamma model (specified parameters were determined by Modeltest as: R A-C 1.46915062, R A-G 5.50077059, R A-T 0.83300576, R C-G 0.72958603, R C-T 9.08816934, rG-t 1.000000, gamma shape = 1.476644, pinvar = 0.608789) in PAUP* 4.0a build 169 (Swofford 2002). Node support was evaluated using 1000 bootstrap replicates. MrBayes 3.2.7 [75,76] was run at the CIPRES Science Gateway V.3.3 (https://www.phylo.org/, accessed on 30 July 2021). The data were partitioned into first, second, and third codon positions, each with their own unlinked GTR+I+gamma rate matrix. Two independent runs of 10,000,000 generations were used to output trees every 1000 generations. The initial 25% of trees was discarded in each run as burn in, and the remaining trees were used to generate a majority rule consensus tree.

### 5.5. High Pressure Freeze Fixation

Zygospores and filaments of *S. mirabilis* were high-pressure frozen and freeze substituted (HPF/FS) according to Aichinger and Lütz-Meindl [77]. Briefly, field samples enriched in zygospores were fixed with a LEICA EMPACT high pressure freezer and freeze substituted in a Leica EM AFS FS apparatus (Leica Microsystems GmbH, Vienna, Austria), either in 1% OsO_4_ (in the case of immuno-stainings) or in 2% OsO_4_ and 0.05% uranyl acetate in acetone at −80 °C for 60 h, temperature raised to −30 °C within 5 h (10 °C/h), maintained at −30 °C for 4 h, temperature raised to 20 °C within 20 h (2.5 °C/h). Samples were either embedded in an Agar Low viscosity resin kit (Agar Scientific, Essex, UK), for 0.3% toluidine blue staining (semi-thin sections) and TEM observations, or in LR-white (medium grade, Science Services, Munich, Germany) for immunocytochemistry.

### 5.6. Transmission Electron Microscopy

Ultrathin sections of HPF/FS material were prepared with a Reichert Ultracut (Leica Microsystems, Wien, Austria), counterstained with 2% uranyl acetate and Reynold’s lead citrate. The samples were observed using a Zeiss Libra 120 transmission electron microscope (Carl Zeiss AG, Oberkochen Germany) at 80 kV, which was equipped with a 2 × 2k digital high speed camera and operated by ImageSP software (Albert Tröndle Restlichtverstärker Systeme, Moorenweis, Germany). 

### 5.7. Cell Wall Extraction and Glycan Micro Array Analysis

*Spirogyra* sp. samples collected in the field and containing filaments and zygospores were freeze dried (CoolSafe 15L freeze dryer; LaboGene A/S, Allerød, Denmark) and their cell walls, i.e., alcohol-insoluble residue (AIR), were extracted in 75% ethanol until the supernatant was transparent. Subsequently, cell wall polysaccharides and glycoproteins were sequentially extracted from AIR in 50 mM CDTA (solubilizing pectins, glycoproteins) and 4 M NaOH (solubilizing hemicelluloses, glycoproteins). The following glycan microarray analysis [78] was performed as previously described in detail [23,79]. Arrays were printed with an ArrayJet Sprint microarray printer (ArrayJet, Roslin, UK) as distinct dots onto nitrocellulose membranes and probed with 46 cell wall probes, including antibodies, carbohydrate binding modules and lectins (Supplementary Table S3). The probes were diluted as follows: 1:10 (JIM and LM probes, MAC207), 1:200 (INRA-RU1+2, 2F4, lectins), 1:1000 (BS-400 probes, CBMs). For controls, primary antibodies were heat-inactivated prior to use. The microarray analysis was repeated three times.

### 5.8. Immunostaining of Selected Cell Wall Components

Semi-thin sections (0.6–1.5 µm) of HPF/FS material (1% OsO_4_, 0.05% uranyl acetate, embedded in LR-white, see above) were immobilized on 10 well microscope slides and blocked with phosphate-buffered saline (PBS) containing 2% (*w*/*v*) BSA and 0.05% Tween-20 (*v*/*v*) for 45 min. The droplets were then removed with filter paper and samples were rinsed 3× with PBS using an insulin syringe. Subsequently, samples were incubated with a primary antibody (1:20 in PBS+0.1% Tween20) at 4°C over night: JIM13 (binding to AGP), LM19 (partially methylesterified homogalacturonan; HG), JIM5 (HG with low degree of esterification (DE)), LM15 (Xyloglucan XXXG motif), LM25 (xyloglucan), BS-400-4 ((1->4)-β-D-mannan), LM1 (extensin), LM6 ((1->5)α-L-arabinan), LM10 ((1->4)-β-D-xylan)). For a comprehensive list of antibodies and manufacturers see also Supplementary Table S4. The primary antibody was removed with filter paper and the samples washed with PBS (3 × 10 min). Cells were then incubated with a secondary antibody for 3 h in the dark (1:80 in PBS; goat anti-rat IgM (μ-chain specific)—FITC conjugate, goat anti-rat IgG—FITC conjugate, goat anti-mouse lgG—FITC conjugate). After washing with PBS as described above, samples were mounted in PBS. For controls, the primary antibody was omitted.

### 5.9. Confocal Raman Spectroscopy

Zygospores from field samples of *Spirogyra* were transferred to glass slides. A coverslip was placed on top and sealed with nail polish. On the prepared slides we performed Confocal Raman microscopy using an Alpha300RA (WITec GmbH, Germany), as previously described by Permann et al. [23]. In this work, the laser (excitation wavelength of 532 nm) power was set to 5 mW. At different focal positions (z-stack) images were acquired by collecting spectra with a 0.05 s integration time and moving the sample in increments of 0.3 µm. For detailed measurements, the laser power was increased to 20 mW and the integration time was set to 0.1 s. Project FIVE Plus (WITec GmbH, Germany) was used for spectral processing and data analysis. After cosmic ray removal and baseline correction, different peak positions were integrated to display the chemical distribution maps. Additionally, *Lycopodium* spores were purchased from Sigma Aldrich (Austria) and measured with the same Raman microscope. The extracted average spectrum from the *Lycopodium* spore wall was added to the reference compound database (*n* = ~250). To obtain detailed insights into the composition of the *Spirogyra* cell wall we fitted the extracted average spectra of the cell wall as a linear combination of the measured reference spectra using the Orthogonal Matching Pursuit method [53].

## Data Availability

The newly generated sequences were deposited under accession numbers MZ813178 and MZ813179 to NCBI.

## Supplementary Materials

The following Supplementary Figures and Tables are available online at https://www.mdpi.com/article/10.3390/plants10081740/s1, Figure S1: Isolated culture of Spirogyra mirabilis with zygospores produced under laboratory conditions. Precultivated on BBM (Bold’s Basal Medium) under a 16/8 h light-dark regime, 20/15 °C, and ~30 μmol photons m^−2^ s^−1^ PAR, conjugation conditions: APW (Artificial Pond Water) at 184 μmol photons m^−2^ s^−1^ and continuous light. Scale bar 0.5 mm, Figure S1: Resulting phylogenetic tree from the maximum likelihood analysis of *rbc*L and *atp*B sequences from *Spirogyra* sp. (Kühtai) (arrow) in the context of other sequenced *Spirogyra* species. The tree is oriented, and the major clades are labeled, after Stancheva et al. [27] and Takano et al. [38]. Morphologically similar *S. longata* indicated by arrowhead. Node support values correspond to ML bootstrap/BPP values. Scale bar indicates the expected number of substitutions/sites, Figure S3: Fluorescent images of S. mirabilis zygospores. (a) JIM13 (AGP), (b) LM19 (Partially methylesterified HG ), (c) LM15 (Xyloglucan (XXXG motif)), (d) BS-400-4 ((1->4)-β-D-mannan), (e) LM1 (Extensin), (f) LM25 (Xyloglucan), (g) LM6 ((1->5)α-L-arabinan), (h) JIM5 (HG with low degree of esterification), (i) LM10 ((1->4)-β-D-xylan), (j) control image (primary antibody was omitted) for JIM13, LM19, LM1, LM25, (k) control image (primary antibody was omitted) for LM15, LM6, JIM5, LM10, (l) control image (primary antibody was omitted) for BS-400-4. Scale bars 20 μm, Figure S4: (a) Average spectrum extracted from the released cell content shows mainly a lipid character (peaks CH, 1654, and 1486 cm^−1^). (b) Reference spectra chosen by the linear combination of reference spectra from a database using the Orthogonal Matching Pursuit Method. Here the *Lycopodium* spore cell wall spectrum shows the highest contribution to the outer layer, followed by the flavanone-7-O-glycoside naringin, linoleic acid, and kaempferol (also flavanone). The *Lycopodium* pollen spore image is based on the integration of the aromatic peak at 1604 cm^−1^, see spectrum. The image is a projection of six measurements (z-stack scan) using a sum filter. (c) The fitted reference spectra for the chemical composition of the inner layer. Here, the cellulose has the highest contribution, followed by aromatic compounds and unsaturated aliphatic alcohols, Table S1: Comparison of the morphological characters of the isolate with *S. longata* (clade I), *S. mirabilis* (clade IV), and *S. pratensis* (clade IV) on the basis of Kadlubowska [22], Prescott [58], Pascher [57] and Takano et al. [38], Table S2: List of published *rbcL* and *atpB* gene sequences included in the present phylogenetic analysis, Table S3 Cell wall probes used in the present study for CoMPP analysis, Table S4 Antibodies used in the present study.

## Abbreviations

AGP	arabinogalactan protein
APW	artificial pond water
BBM	Bold’s basal medium
BI	Bayesian inference
HG	Homogalacturonan
ML	maximum likelihood
PAR	photosynthetically active radiation
PBS	phosphate-buffered saline
RGI	rhamnogalacturonan I
TEM	transmission electron microscopy
