# Supplementary material for: Induction of Conjugation and Zygospore Cell Wall Characteristics in the Alpine Spirogyra mirabilis (Zygnematophyceae, Charophyta): Advantage under Climate Change Scenarios?
Source: Plants (Basel). 2021 Aug 23;10(8):1740. doi: 10.3390/plants10081740 (PMC8402014; doi:10.3390/plants10081740)
Supplement: Supplementary file 1 [file plants-10-01740-s001.zip › ESM 1_final.pdf]

Supplementary Figures:

Induction of conjugation and zygospore cell wall characteristics in the alpine *Spirogyra mirabilis* (Zygnematophyceae, Charophyta): Advantage under climate change scenarios?

Plants

Charlotte Permann[1], Klaus Herburger[2], Martin Felhofer[3], Notburga Gierlinger[3], Louise A. Lewis[4], Andreas Holzinger[1*]

*Author for correspondence: Andreas.Holzinger@uibk.ac.at
Department of Botany, University of Innsbruck, Functional Plant Biology, 6020 Innsbruck, Austria

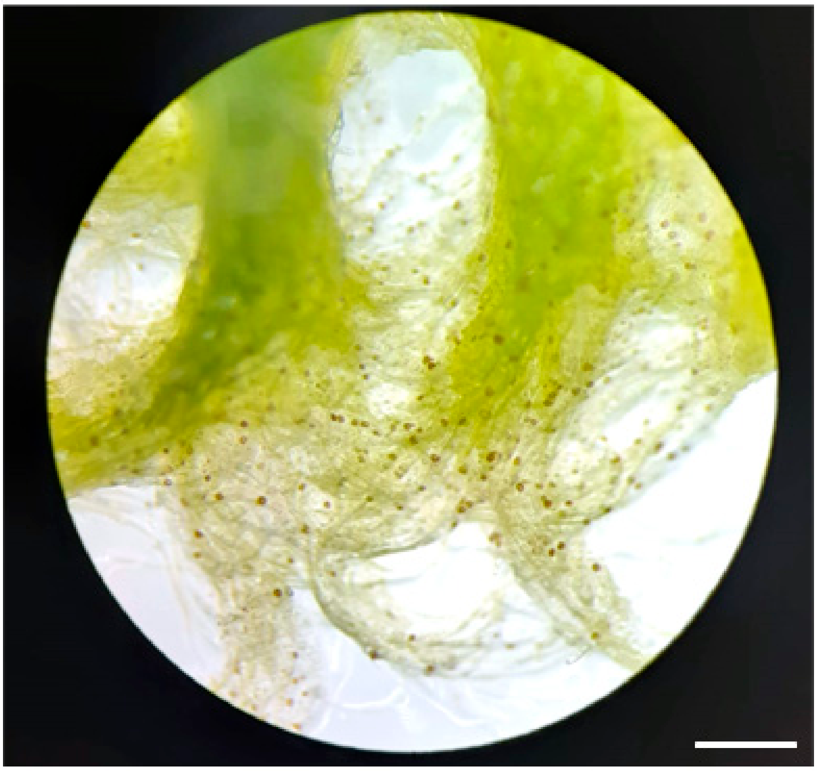

Suppl. Fig. S1 Isolated culture of *Spirogyra mirabilis* with zygospores produced under laboratory conditions. Precultivated on BBM (Bold's Basal Medium) at a 16/8 h light-dark regime, 20/ 15 °C and ~ 30 µmol photons m^{-2} s^{-1} PAR, conjugation conditions: APW (Artificial Pond Water) at 184 µmol photons m^{-2} s^{-1} and continuous light. Scale bar 0.5 mm

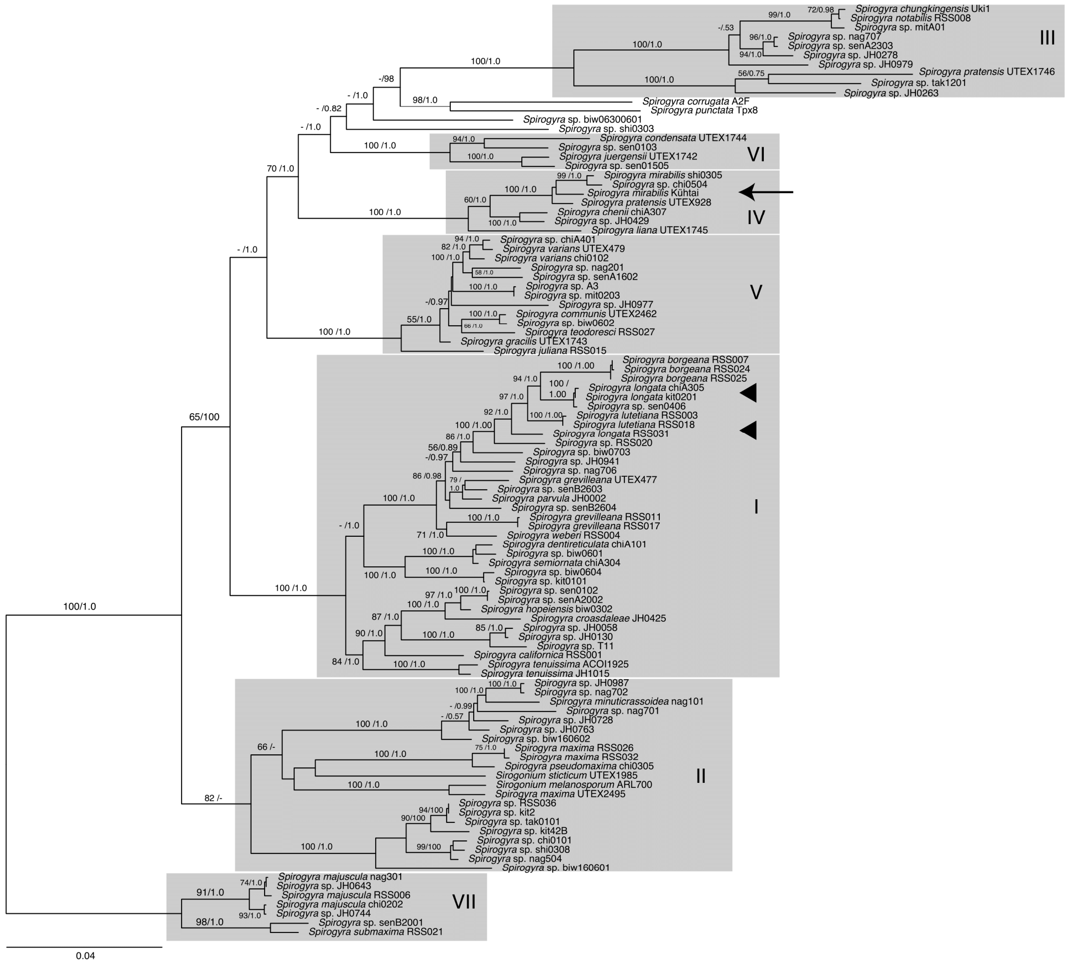

Suppl. Fig. S2 Resulting phylogenetic tree from the maximum likelihood analysis of *rbc*L and atpB sequences from Spirogyra sp. (Kühtai) (arrow) in the context of other sequenced Spirogyra species. The tree is oriented, and the major clades are labeled, after Stancheva et al. (2013) and Takano et al. (2019). Morphologically similar S. longata indicated by arrowhead. Node support values correspond to ML bootstrap/BPP values. Scale bar indicates the expected number of substitutions/ sites.

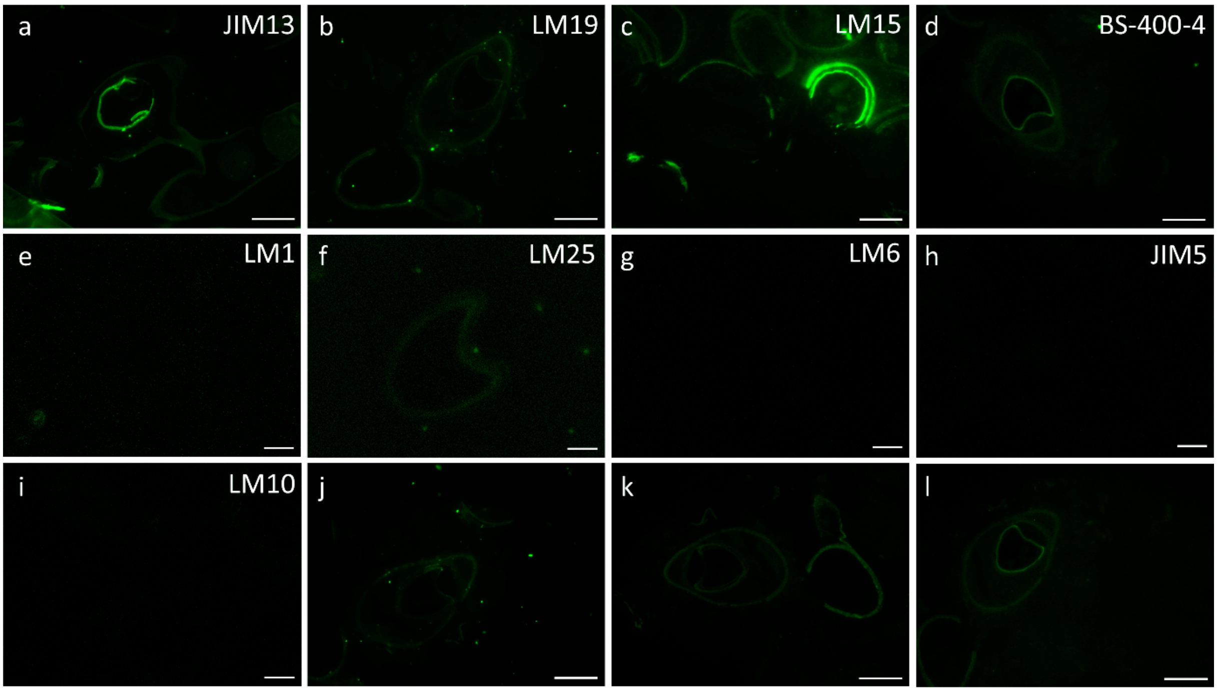

Suppl. Fig. S3 Fluorescent images of S. mirabilis zygospores. (a) JIM13 (AGP), (b) LM19 (Partially methylesterified HG), (c) LM15 (Xyloglucan (XXXG motif)), (d) BS-400-4 ((1->4)- β -D-mannan), (e) LM1 (Extensin), (f) LM25 (Xyloglucan), (g) LM6 ((1->5)α-L-arabinan), (h) JIM5 (HG with low degree of esterification), (i) LM10 ((1->4)-β-D-xylan), (j) control image (primary antibody was omitted) for JIM13, LM19, LM1, LM25, (k)) control image (primary antibody was omitted) for LM15, LM6, JIM5, LM10, (l) control image (primary antibody was omitted) for BS-400-4. Scale bars 20 µm

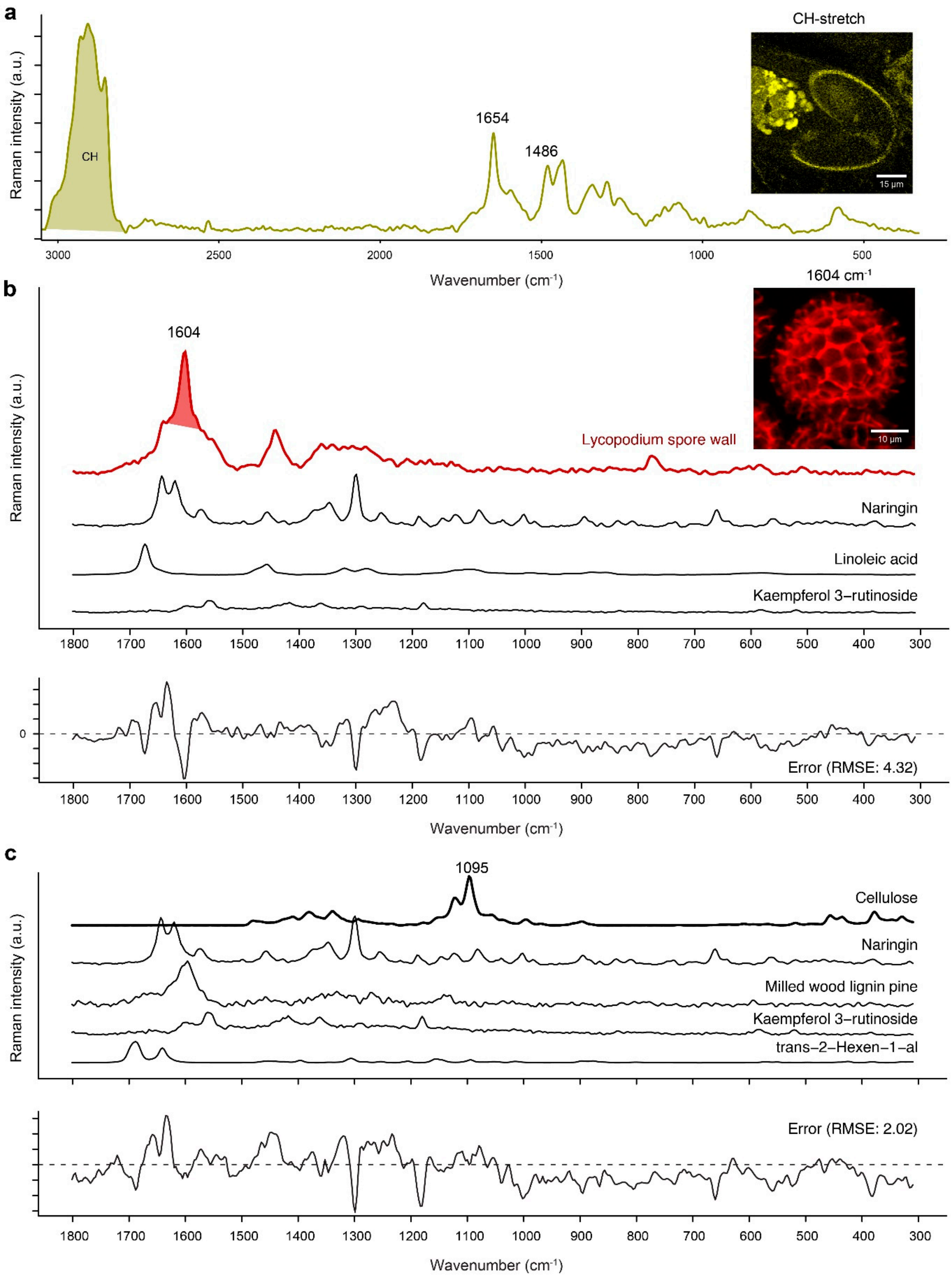

Suppl. Fig. S4 (a) Average spectrum extracted from the released cell content shows mainly a lipid character (peaks CH, 1654, and 1486 cm-1). (b) Reference spectra chosen by the linear combination of reference spectra from a database using the Orthogonal Matching Pursuit Method. Here the Lycopodium pollen spore cell wall spectrum shows the highest contribution to the outer layer, followed by the flavanone-7-O-glycoside Naringin, Linoleic acid, and Kaempferol (also flavanone). The Lycopodium pollen spore image is based on the integration of the aromatic peak at 1604 cm-1, see spectrum. The image is a projection of six measurements (z-stack scan) using a sum filter. (c) The fitted reference spectra for the chemical composition of the inner layer. Here, the cellulose has the highest contribution, followed by aromatic compounds, and unsaturated aliphatic alcohols.
