# Supplementary material for: Induction of Conjugation and Zygospore Cell Wall Characteristics in the Alpine Spirogyra mirabilis (Zygnematophyceae, Charophyta): Advantage under Climate Change Scenarios?
Source: Plants (Basel). 2021 Aug 23;10(8):1740. doi: 10.3390/plants10081740 (PMC8402014; doi:10.3390/plants10081740)
Supplement: Supplementary file 1 [file plants-10-01740-s001.zip › ESM 2_final.pdf]

Supplementary Tables:

Induction of conjugation and zygospore cell wall characteristics in the alpine *Spirogyra mirabilis* (Zygnematophyceae, Charophyta): Advantage under climate change scenarios?

Plants

Charlotte Permann[1], Klaus Herburger[2], Martin Felhofer[3], Notburga Gierlinger[3], Louise A. Lewis[4], Andreas Holzinger[1*]

*Author for correspondence: Andreas.Holzinger@uibk.ac.at
Department of Botany, University of Innsbruck, Functional Plant Biology, 6020 Innsbruck, Austria

Table S1. Comparison of the morphological characters of the isolate with *Spirogyra longata* (clade I), *Spirogyra mirabilis* (clade IV) and *Spirogyra pratensis* (clade IV) on the basis of Kadlubowska (1984), Prescott (1951), Pascher (1913) and Takano et al. (2019).

		Kadlubowska (1984)			Prescott (1951)			Pascher (1913)		Takano et al. (2019)	
	S. sp. (Kühtai)	*S. longata*	*S. mirabilis*	*S. pratensis*	*S. longata*	*S. mirabilis*	*S. pratensis*	*S. longata*	*S. mirabilis*	*S. longata*	*S. mirabilis*
**Width**	23-34 µm	26-32 µm	21-33 µm	17-20 µm	20-36 µm	24-26-(27) µm	17-20 µm	28-38 µm	18-27 µm	26-32 µm	20-26 µm
**Length**	up to 5x length	-	-	-	Up to 10x length	Up to 10x length	80-95-(240) µm	2-10x	4-10x	110-260 µm	105-270 µm
**End wall**	plane	plane	plane	plane	plane	plane	plane	plane	plane	plane	plane
**Chloroplasts/ cell**	1	1	1	1(2)	1	1	1 (2)	1	1	1	1
**Turns/ cell**	2-4	-	-	-	2-5	4-7	1-8	2-5	4-7	3-6	6-8
**Conjugation type**	scalariform/ lateral	scalariform/ lateral	scalariform	scalariform/ lateral	-	-	-	-	-	scalariform	-
**Conjugation tube formation**	both gametangia	both gametangia	both gamtangia	both gametangia	both gametangia	both gametangia	both gametangia	-	-	both gametangia	-
**Vegetative cell type**	not inflated	not inflated	not inflated	very inflated (>60)	not inflated	not inflated	not inflated	not inflated	-	not inflated	-
**Gametangia shape**	inflated	not inflated	inflated	inflated (>38)	cylindrical	inflated	inflated	not inflated	-	cylindrical	-
**Zygospore shape**	polymorph	ellipsoid-cylindric	ellipsoid	ellipsoid	(elongate)-ovate	ovate/ ellipsoid	ellipsoid/ subcylindrical -ovate	ovate/ flat ends	-	ovate	-
**Zygospore width**	27-37 µm	25-35 µm	-	24-36 µm	30-(38) µm	24-29 µm	24-36 µm	28-37 µm	-	28-32 µm	-
**Zygospore length**	32-61 µm	46-100 µm	-	50-70 µm	50-(83) µm	50-83 µm	50-60-(70) µm	56-74 µm	-	48-74 µm	-
**Me surface**	smooth	smooth	smooth	smooth	smooth	smooth	smooth	-	-	smooth	-
**Me colour**	(yellow)-brown	brown	yellow-brown	yellow	brownish	brown	yellow	light yellow	-	yellow	-
**Suture**	no	yes	yes	-	yes	no	no	-	-	-	-
**Points**	30/ 30	18/ 26	17/ 22	13/ 24	20/ 28	22/ 28	19/ 28	14/ 22	9/ 10	18/ 28	7/ 10
**Points (%)**	100	69.23	77.27	54.17	71.43	78.57	67.86	63.64	90	64.29	70
**Points average (%)**		67.15	**79.00**	61.02	67.15	**79.00**	61.02	67.15	**79.00**	67.15	**79.00**
**Points Ranking**		2	**1**	3	2	**1**	3	2	**1**	2	**1**
**Nr. of misses**		2/ 13	1/ 11	3/ 12	2/ 14	1/ 14	2/ 14	2/ 11	0/ 5	2/ 14	1/ 5
**Nr. of misses (%)**		15.38	9.1	25	14.29	7.14	14.29	18.18	0	14.29	20
**Misses average (%)**		15.54	**9.06**	19.65	15.54	**9.06**	19.65	15.54	**9.06**	15.54	**9.06**
**Misses Ranking**		2	**1**	3	2	**1**	3	2	**1**		**1**

Point System

dark green	☐	=	match	=	2P
light green	☐	=	light match	=	1P
white	☐	=	no information	=	0P
red	☐	=	no match	=	-1P

Table S2. List of published *rbc*L and *atp*B gene sequences included in the present phylogenetic analysis.

Strain	Species	*rbcL*	*atpB*
ARL 700	*Sirogonium melanosporum* (Randhawa) Transeau	L13484	KC779088
UTEX 1985	*Sirogonium sticticum* (Sm.) Kütz.	DQ015924	KC779089
RSS007	*Spirogyra borgeana* Transeau	KC779194	KC779128
RSS024	*Spirogyra borgeana* Transeau	KC779209	KC779138
RSS025	*Spirogyra borgeana* Transeau	KC779210	KC779139
RSS001	*Spirogyra californica* Stancheva, J. D. Hall, McCourt et Sheath	KC779188	KC779123
Uki1 [=NIES-4302]	*Spirogyra chungkingensis* Jao JPS001	MK558136	MK558167
UTEX 2462	*Spirogyra communis* (Hassall) Kütz.	DQ015932	KC779090
UTEX 1744	*Spirogyra condensata* (Vaucher) Kütz.	DQ015936	KC779091
JH0425	*Spirogyra croasdaleae* Blum	KC779170	KC779107
A2F [=NIES-4303]	*S. corrugata* Transeau JPS002	MK558126	MK558170
RSS033	*Spirogyra fluviatilis* Hilse	KC779218	KC779146
UTEX 1743	*Spirogyra gracilis* (Hassall) Kütz.	DQ015937	KC779092
RSS011	*Spirogyra grevilleana* (Hassall) Kütz.	KC779197	KC779130
RSS017	*Spirogyra grevilleana* (Hassall) Kütz.	KC779202	KC779133
UTEX 477	*Spirogyra grevilleana* (Hassall) Kütz.	DQ015938	KC779093
UTEX 1742	*Spirogyra juergensii* Kütz.	DQ015939	KC779094
RSS015	*Spirogyra juliana* Stancheva, J. D. Hall, McCourt et Sheath	KC779200	KC779131
UTEX 1745	*Spirogyra liana* Transeau	DQ015940	KC779095
RSS031	*Spirogyra longata* (Vaucher) Kütz.	KC779216	KC779144
RSS003	*Spirogyra lutetiana* P. Petit	KC779190	KC779125
RSS018	*Spirogyra lutetiana* P. Petit	KC779203	KC779134
RSS006	*Spirogyra majuscula* Kütz.	KC779193	KC779127
RSS026	*Spirogyra maxima* (Hassall) Kütz.	KC779211	KC779140

RSS032	*Spirogyra maxima* (Hassall) Kütz.	KC779217	KC779145
UTEX 2495	*Spirogyra maxima* (Hassall) Kütz.	DQ015941	AF408797
RSS008	*Spirogyra notabilis* Taft	KC779195	KC779129
JH0002	*Spirogyra parvula* (Transeau) Czurda	KC779166	KC779101
UTEX 1746	*Spirogyra pratensis* Transeau	DQ015949	KC779098
UTEX 928	*Spirogyra pratensis* Transeau	DQ015948	KC779097
Tpx8 [=NIES-4314]	*S. punctata* Cleve JPS013	MK558127	MK558169
JH0058	*Spirogyra* sp.	DQ015954	KC779102
JH0130	*Spirogyra* sp.	DQ015955	KC779104
JH0263	*Spirogyra* sp.	KC779168	KC779105
JH0278	*Spirogyra* sp.	KC779169	KC779106
JH0429	*Spirogyra* sp.	KC779171	KC779108
JH0643	*Spirogyra* sp.	KC779173	KC779110
JH0728	*Spirogyra* sp.	KC779174	KC779111
JH0744	*Spirogyra* sp.	KC779175	KC779112
JH0763	*Spirogyra* sp.	KC779176	KC779113
JH0941	*Spirogyra* sp.	KC779179	KC779115
JH0977	*Spirogyra* sp.	KC779182	KC779118
JH0979	*Spirogyra* sp.	KC779183	KC779119
JH0987	*Spirogyra* sp.	KC779185	KC779121
RSS020	*Spirogyra* sp.	KC779205	KC779135
RSS036	*Spirogyra* sp.	KC779220	KC779148
T11	*Spirogyra* sp. JPS050	MK558113	MK558151
mit0203	*Spirogyra* sp. JPS030	MK558104	MK558176
mitA01	*Spirogyra* sp. JPS031	MK558135	MK558186
A3	*Spirogyra* sp. JPS016	MK558103	MK558175

senA1602	*Spirogyra* sp. JPS038	MK558108	MK558180
senA2002	*Spirogyra* sp. JPS039	MK558111	MK558150
senA2303	*Spirogyra* sp. JPS040	MK558138	MK558172
senB2001	*Spirogyra* sp. JPS041	MK558142	MK558185
senB2603	*Spirogyra* sp. JPS042	MK558120	MK558155
senB2604	*Spirogyra* sp. JPS043	MK558122	MK558145
sen0102	*Spirogyra* sp. JPS044	MK558110	MK558149
sen0103	*Spirogyra* sp. JPS045	MK558129	MK558181
sen01505	*Spirogyra* sp. JPS046	MK558130	MK558191
sen0406	*Spirogyra* sp. JPS047	MK558125	MK558156
kit0101	*Spirogyra* sp. JPS027	MK558118	MK558154
RSS021	*Spirogyra submaxima* Transeau	KC779206	KC779136
ACOI 1925	*Spirogyra tenuissima (Hassall)* Kütz.	KC779187	KC779099
JH1015	*Spirogyra tenuissima (Hassall)* Kütz.	KC779186	KC779122
RSS027	*Spirogyra teodoresci* Transeau	KC779212	KC779141
UTEX 479	*Spirogyra varians* (Hassall) Kütz.	DQ015951	KC779100
RSS004	*Spirogyra weberi* Kütz.	KC779191	KC779126

Table S3. Cell wall probes used in the present study

No.	Polymer class	Code	Epitope	Source	Reference
1	Pectin	2F4	Ca^{2+} cross linked HG	PlantProbes	Liners et al. 1989
2		JIM5	Homogalactauronan with a low DE	PlantProbes	Willats et al. 2000
3		JIM7	Homogalactauronan with a high DE	PlantProbes	Willats et al. 2000
4		LM19	Methylesterified homogalactauronan (low DE)	PlantProbes	Verhertbruggen et al. 2009
5		LM20	Methylesterified homogalactauronan (high DE)	PlantProbes	Verhertbruggen et al. 2009
6		INRA-RU2	Backbone of rhamnogalacturonan I (4 units)	INRA	Ralet et al. 2010
7		INRA-RU1	Backbone of rhamnogalacturonan I (12 units)	INRA	Ralet et al. 2010
8		LM5	(1→4)-β-D-galactan	PlantProbes	Jones et al. 1997
9		LM6	(1→5)-α-L-arabinan	PlantProbes	Willats et al. 1998
10		LM13	Linearised (1→5)-α-L-arabinan	PlantProbes	Moller et al. 2008
11		LM16	(1→5)-α-L-arabinan, RG backbone	PlantProbes	Verhertbruggen et al. 2009
12		LM8	Xylogalacturonan	PlantProbes	Willats et al. 2004
13	Xyloglucan	LM15	Xyloglucan (XXXG motif)	PlantProbes	Marcus et al. 2008
14		LM24	Xyloglucan (XLLG motiv, weak to XXLG)	PlantProbes	Pedersen et al. 2012
15		LM25	Xyloglucan (XXXG, XXLG, XLLG motiv; also GGGGGG)	PlantProbes	Pedersen et al. 2012
16	Xylan	LM10	(1→4)-β-D-xylan (low-substituted)	PlantProbes	McCartney et al. 2005
17		LM11	(1→4)-β-D-xylan (low-substituted)/arabinoxylan)	PlantProbes	McCartney et al. 2005
18		LM23	(1→4)-β-D-xylan/xylogalacturonan	PlantProbes	Pedersen et al. 2012
19	β-glucans	BS-400-2	(1→3)-β-D-glucan	Biosupplies	Meikle et al. 1991
20		BS-400-3	(1→3) (1→4)-β-D-glucan	Biosupplies	Meikle et al. 1994
21	Mannans	BS-400-4	(1→4)-β-D-mannan	Biosupplies	Pettolino et al. 2001
22		LM21	(1→4)-β-D-mannan/galactomannan/glucomannan	PlantProbes	Marcus et al. 2010
23		LM22	(1→4)-β-D-mannan/glucomannan	PlantProbes	Marcus et al. 2010
24	AGPs	JIM8	AGP (Gal-rich)	PlantProbes	McCabe et al. 1997
25		JIM13	β-GlcA-(1→3)-α-GalA-(1→2)-α-Rha)	PlantProbes	Knox et al. 1991
26		LM2	AGP (1→6)-β-Gal with terminal β-GlcA)	PlantProbes	Smallwood et al. 1996
27		LM14	AGP and/or pectic type II arabinogalactan	PlantProbes	Moller et al. 2008
28		MAC207	AGP (β-GlcA-(1→3)-α-GalA-(1→2)-α-Rha)	PlantProbes	Pennell. et al. 1989
29	Extensins	LM1	Extensin (hydroxyproline-rich motif THRGP)	PlantProbes	Smallwood et al. 1995
30		LM3	Extensin	PlantProbes	Feng et al. 2014
31		JIM11	Extensin	PlantProbes	Smallwood et al. 1994
32		JIM12	Extensin	PlantProbes	Smallwood et al. 1994
33		JIM19	Extensin	PlantProbes	Smallwood et al. 1994
34		JIM20	Extensin	PlantProbes	Smallwood et al. 1994
35	Cellulose	CBM3a	Crystalline cellulose	PlantProbes	Blake et al. 2006

36		CBM2a	Crystalline cellulose	PlantProbes	McLean et al. 2000
37	Lectins	B-1005	Concanavalin A; α-linked Man	Vector Laboratories	Yue et al. 2009
38		B-1015	Soybean agglutinin; α- or β-linked GalNAc	Vector Laboratories	Yue et al. 2009
39		B-1025	Wheat germ agglutinin; dimers and trimers of GlcNAc	Vector Laboratories	Yue et al. 2009
40		B-1065	*Ulex Europaeus* Agglutinin I; α-linked Fuc	Vector Laboratories	Yue et al. 2009
41		B-1085	*Ricinus communis* agglutinin; Gal or GalNAc residues	Vector Laboratories	Yue et al. 2009
42		B-1285	*Bauhinia purpurea* lectin; Gal-α-(1→3) GalNAc, terminal GalNAc	Vector Laboratories	Yue et al. 2009
43		B-1305	*Sambucus nigra* lectin; sialic acid attached to term. Gal, GalNAc	Vector Laboratories	Yue et al. 2009
44		B-1335	*Euonymus europaeus* lectin; Gal-α-(1→3)-Fuc-α-(1→2)-Gal	Vector Laboratories	Opitz et al. 2007
45		B-1365	*Psophocarpus tetragonolobus* lectin; GalNAc	Vector Laboratories	McCarter et al. 2013
46		B-1405	*Psophocarpus tetragonolobus* lectin II; Gal and GalNAc	Vector Laboratories	Barkhordari et al. 2004

Supporting references

Liners F, Letesson JJ, Didembourg C, et al. (1989) Monoclonal antibodies against pectin: recognition of a conformation induced by calcium. Plant Physiol 1:1419-1424.

Barkhordari A, Stoddart RW, McClure SF, et al. (2004) Lectin histochemistry of normal human lung. J Mol Histol 35:147-156.

Blake AW, McCartney L, Flint JE, et al. (2006) Understanding the biological rationale for the diversity of cellulose-directed carbohydrate-binding modules in prokaryotic enzymes. J Biol Chem 281:29321-29329.

Feng T, Nyffenegger C, Højrup P, et al. (2014) Characterization of an extensin-modifying metalloprotease: N-terminal processing and substrate cleavage pattern of *Pectobacterium carotovorum* Prt1. Appl Microbiol Biotechnol 98:10077-10089.

Jones L, Seymour GB, Knox JP (1997) Localization of pectic galactan in tomato cell walls using a monoclonal antibody specific to (1 [->] 4)-[beta]-D-galactan. Plant Physiol 113:1405-1412.

Knox JP, Linstead PJ, Cooper JPC, et al. (1991) Developmentally regulated epitopes of cell surface arabinogalactan proteins and their relation to root tissue pattern formation. Plant J 1:317-326.

Marcus SE, Blake AW, Benians TA, et al. (2010) Restricted access of proteins to mannan polysaccharides in intact plant cell walls. Plant J 64:191-203.

Marcus SE, Verhertbruggen Y, Hervé C, et al. (2008) Pectic homogalacturonan masks abundant sets of xyloglucan epitopes in plant cell walls. BMC Plant Biol 8:1-12.

McCabe PF, Valentine TA, Forsberg LS, et al. (1997) Soluble signals from cells identified at the cell wall establish a developmental pathway in carrot. Plant Cell 9:2225-2241.

McCarter C, Kletter D, Tang H, et al. (2013) Prediction of glycan motifs using quantitative analysis of multi-lectin binding: M otifs on MUC 1 produced by cultured pancreatic cancer cells. Proteomics Clin Appl 7:632-641.

McCartney L, Marcus SE, Knox JP (2005) Monoclonal antibodies to plant cell wall xylans and arabinoxylans. J Histochem Cytochem 53:543-546.

McLean BW, Bray MR, Boraston AB, et al. (2000) Analysis of binding of the family 2a carbohydrate-binding module from *Cellulomonas fimi* xylanase 10A to cellulose: specificity and identification of functionally important amino acid residues. Protein Eng 13:801-809.

Meikle PJ, Bonig I, Hoogenraad NJ, Clarke AE et al. (1991) The location of (1→ 3)-β-glucans in the walls of pollen tubes of *Nicotiana alata* using a (1→ 3)-β-glucan-specific monoclonal antibody. Planta 185:1-8.

Meikle PJ, Hoogenraad NJ, Bonig I et al. (1994) A (1→ 3, 1→ 4)-β-glucan-specific monoclonal antibody and its use in the quantitation and immunocytochemical location of (1→ 3, 1→ 4)-β-glucans. Plant J 5:1-9.
Moller I, Marcus SE, Haeger A, et al. (2008) High-throughput screening of monoclonal antibodies against plant cell wall glycans by hierarchical clustering of their carbohydrate microarray binding profiles. Glycoconj J 25:37-48.
Opitz L, Salaklang J, Büttner H, et al. (2007) Lectin-affinity chromatography for downstream processing of MDCK cell culture derived human influenza A viruses. Vaccine 25:939-947.
Pedersen HL, Fangel JU, McCleary B, et al. (2012) Versatile high resolution oligosaccharide microarrays for plant glycobiology and cell wall research. J Biol Chem 287:39429-38.
Pennell RI, Knox JP, Scofield GN et al. (1989) A family of abundant plasma membrane-associated glycoproteins related to the arabinogalactan proteins is unique to flowering plants. J Cell Biol 108:1967-1977.
Pettolino FA, Hoogenraad NJ, Ferguson C, et al. (2001) A (1→ 4)-β-mannan-specific monoclonal antibody and its use in the immunocytochemical location of galactomannans. Planta 214:235-242.
Ralet MC, Tranquet O, Poulain D, et al. (2010) Monoclonal antibodies to rhamnogalacturonan I backbone. Planta 231:1373-1383.
Smallwood M, Beven A, Donovan N, et al. (1994) Localization of cell wall proteins in relation to the developmental anatomy of the carrot root apex. Plant J 5:237-246.
Smallwood M, Martin H, Knox JP (1995) An epitope of rice threonine-and hydroxyproline-rich glycoprotein is common to cell wall and hydrophobic plasma-membrane glycoproteins. Planta 196:510-522.
Smallwood M, Yates EA, Willats WG, et al. (1996) Immunochemical comparison of membrane-associated and secreted arabinogalactan-proteins in rice and carrot. Planta 198:452-459.
Verhertbruggen Y, Marcus SE, Haeger A, et al. (2009) An extended set of monoclonal antibodies to pectic homogalacturonan. Carbohydr Res 344:1858-1862.
Verhertbruggen Y, Marcus SE, Haeger A, et al. (2009) Developmental complexity of arabinan polysaccharides and their processing in plant cell walls. Plant J 59:413-425.
Willats WGT, Limberg G, Buchholt HC, et al. (2000) Analysis of pectic epitopes recognised by hybridoma and phage display monoclonal antibodies using defined oligosaccharides, polysaccharides, and enzymatic degradation. Carbohydr Res 327:309-320.
Willats WGT, Marcus SE, Knox JP (1998) Generation of a monoclonal antibody specific to (1→ 5)-α-L-arabinan. Carbohydr Res 308:149-152.
Willats WGT, McCartney L, Steele-King CG, et al. (2004) A xylogalacturonan epitope is specifically associated with plant cell detachment. Planta 218:673-681.
Yue T, Goldstein IJ, Hollingsworth MA, et al. (2009) The prevalence and nature of glycan alterations on specific proteins in pancreatic cancer patients revealed using antibody-lectin sandwich arrays. Mol Cell Proteomics 8:1697-1707.

Table S4. Antibodies used in the present study

Primary Antibody					Secondary Antibody	
**Polymere class**	**Code**	**Epitope**	**Source**	**Isotype**	**Conjugate**	**Source**
Pectin	JIM5	HG with low DE	PlantProbes	IgG	FITC	Sigma-Aldrich
	LM6	(1->5)α-L-arabinan	PlantProbes	IgG	FITC	Sigma-Aldrich
	LM19	Partially methylesterified HG	PlantProbes	IgM	Fluorescein	Sigma-Aldrich
Xyloglucan	LM15	Xyloglucan (XXXG motif)	PlantProbes	IgG2c	FITC	Sigma-Aldrich
	LM25	Xyloglucan	PlantProbes	IgM	Fluorescein	Sigma-Aldrich
Xylan	LM10	(1->4)-β-D-xylan	PlantProbes	IgG2c	FITC	Sigma-Aldrich
Mannan	BS-400-4	(1->4)- β -D-mannan	Biosupplies	IgG	FITC	Sigma-Aldrich
Extensin	LM1	Extensin	PlantProbes	IgM	Fluorescein	Sigma-Aldrich
AGPs	JIM13	AGP	PlantProbes	IgM	Fluorescein	Sigma-Aldrich
